# Auditory verbal hallucinations related to altered long-range synchrony of gamma-band oscillations

**DOI:** 10.1038/s41598-017-09253-7

**Published:** 2017-08-21

**Authors:** Saskia Steinmann, Gregor Leicht, Christina Andreou, Nenad Polomac, Christoph Mulert

**Affiliations:** Psychiatry Neuroimaging Branch, Department of Psychiatry and Psychotherapy, University Medical Center Hamburg-Eppendorf, Hamburg, Germany

## Abstract

Our understanding of the neural correlates of auditory-verbal-hallucinations (AVH) has substantially increased during the last few years, but is far from sufficient. One current hypothesis, the interhemispheric miscommunication theory, is based on findings from fMRI, DTI and EEG, but there is only limited evidence so far concerning underlying functional coupling mechanisms. Here we report a 64-channel EEG study using lagged phase synchronization analysis and eLORETA source estimation to examine the functional connectivity between bilateral auditory cortices in the gamma-band in 26 schizophrenia patients (13 with and 13 without AVH) and 26 matched healthy controls (HC) while performing a dichotic listening task. We found a significantly reduced right-ear-advantage (REA) in AVH but not in non-AVH patients compared to HC. The major finding was significantly stronger gamma-band connectivity between bilateral auditory cortices during conscious perception of left (versus right) ear syllables in patients with AVH compared to HC and patients without AVH. A significant positive correlation was found between this connectivity alteration and the AVH symptom score in schizophrenia patients. These findings provide further support for the interhemispheric miscommunication hypothesis of AVH pathophysiology by indicating that aberrant gamma-band coupling between auditory cortices is related to the emergence of AVH in schizophrenia.

## Introduction

**Figure 1:**
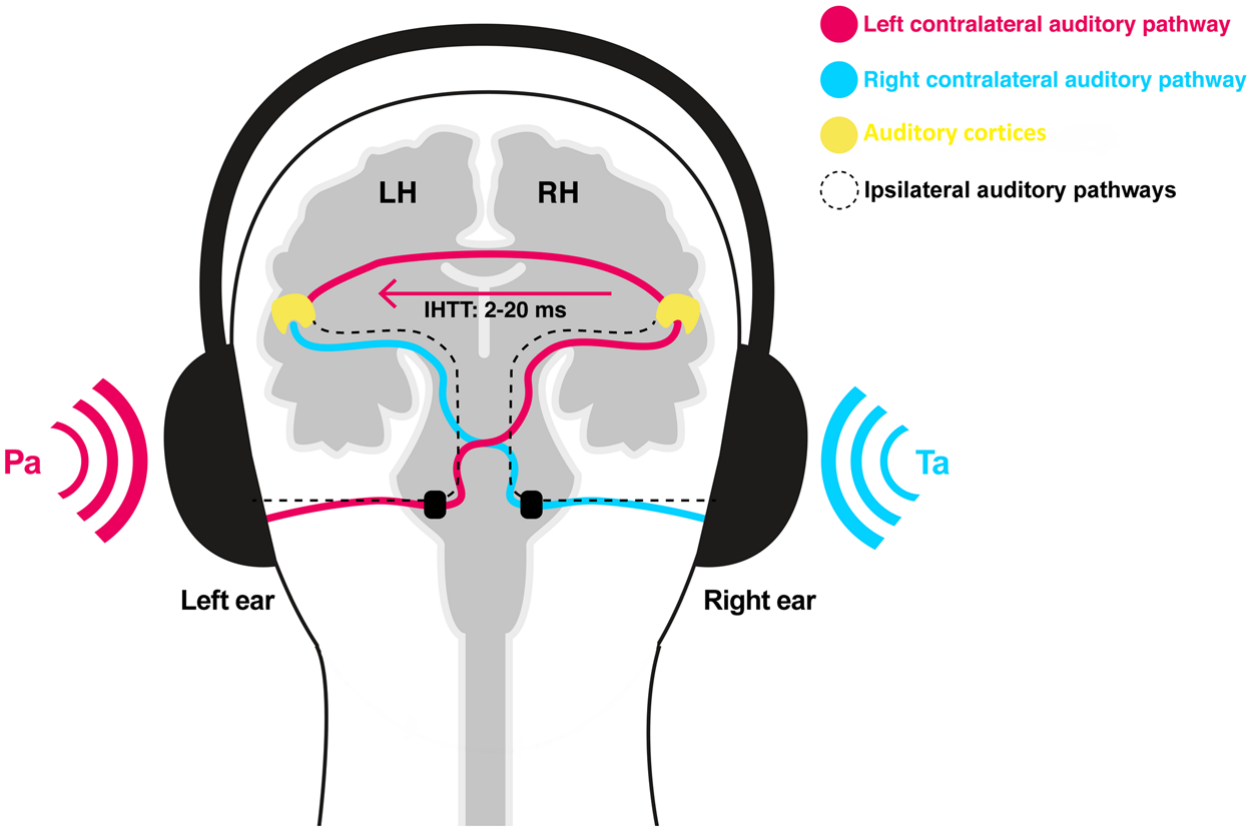
Schematic illustration of the DL procedure. During DL, one syllable is presented to the left ear (PA) and the other one is simultaneously presented to the right ear (TA). Although both ears are connected anatomically via ipsilateral (black dotted lines) and contralateral pathways with (pink and cyan lines) the auditory cortices, ipsilateral pathways are suggested to be blocked during DL. The cyan line indicates the contralateral pathway transmitting the right ear stimulus directly to the speech dominant left hemisphere. In contrast, the syllable running from the left ear to the right hemisphere has to cross the corpus callosum to be efficiently processed (pink line). Yellow: Right and left primary and secondary auditory cortices. LH: Left hemisphere, RH: Right hemisphere. IHTT: Interhemispheric transfer time.

Auditory verbal hallucinations (AVH) – vocal perceptions in the absence of any appropriate external stimulus – are one of the most common positive symptoms in schizophrenia (SCZ). The fact that there is a substantial similarity between AVH and real voices has led researchers to suggest that “hearing voices” are likely to emerge in the context of alterations in a brain network related to speech and language processing^1^. One of the strongest and most often replicated findings for altered speech processing on a behavioral level in hallucinating patients is the disappearance of the right-ear-advantage (REA) during dichotic listening (DL)^2–4^. The DL task is the most frequently used behavioral indicator of language lateralization and typically consists of the presentation of two different verbal stimuli (such as consonant-vowel syllables) at the same time, one to each ear (Fig. 1). Instructed to report the stimulus that is perceived best, healthy right-handed participants more often report the right than the left stimulus, a finding attributed to the supremacy of the contralateral pathways running from the right to the speech-dominant left hemisphere^5^. In contrast, the left ear input initially accesses the right hemisphere and needs additional transfer via interhemispheric pathways before it is processed in the left hemisphere^6^. 95% of right-handers show the typical left-hemispheric language dominance indicating a close link between language lateralization and handedness, and although no mutual genetic factors have been found so far, the two traits show partial pleiotropy^7, 8^.

Our understanding of the neural mechanism underlying the typical REA was strongly improved by recent DTI and EEG studies. For instance, it has been shown that there is striking interindividual variability in the size and shape of interhemispheric pathways in the general population, with stronger pathways improving transcallosal transfer so that individuals reported more stimuli presented to the left ear^9^. Notably, two initial DTI studies demonstrated that the emergence of AVH was associated with stronger interhemispheric auditory pathways in early stages of SCZ^10, 11^, while opposite results were found in chronic SCZ^12–14^. These findings suggest that the structural interhemispheric connectivity may evolve progressively and appear to be associated with patients’ duration of illness. Furthermore, evidence of our own group indicated that Gamma-band Oscillations (GBO) and their synchronization are a key functional mechanism in the transcallosal auditory transfer and play a major role for conscious auditory perception of syllables presented to the left ear^15^. Here, stronger interhemispheric gamma-band coupling between both auditory cortices was associated with a smaller REA. Accordingly, a reduced REA in AVH patients would be in line with stronger interhemispheric gamma-band connectivity. To date, not much is known about alterations in the functional connectivity involved in language processing and AVH, although GBO and their synchronization are a strong candidate given their specific role for perception processes in general^15, 16^ and the growing literature about alterations of GBO in SCZ^17, 18^. While these GBO alterations appear to be complex, an increasing number of studies have reported that particularly positive symptoms of SCZ are correlated with altered oscillatory gamma synchrony in circumscribed sensory brain areas^19–22^. Of interest, one EEG study reported a significant positive correlation between AVH symptom score and interhemispheric gamma-band coupling between primary auditory cortices during an auditory-steady-state-response (ASSR) task^23^, although the ASSR task is not specifically designed to investigate interhemispheric interaction. In contrast, the DL task used in this study is perfectly suited to investigate interhemispheric (mis-)communication, altered auditory perception, and interhemispheric gamma-band coupling in relation to the emergence of AVH, as evidence of our own group indicated that synchronized GBO are crucially involved in transcallosal auditory information transfer^15^.

Hence, based on multimodal evidence from fMRI, DTI and EEG, the interhemispheric miscommunication hypothesis of AVH proposes that altered interhemispheric connectivity between bilateral auditory cortices is related to the emergence of AVH in SCZ. It is widely acknowledged that interhemispheric integration of information is crucial for normal speech processing and comprehension^24^, so that disturbances could give rise to aberrant processing in an auditory network, which might lead to the experience of AVH. In the context of DL, previous studies have shown that stronger interhemispheric pathways as well as stronger gamma-band coupling between bilateral auditory cortices were both associated with a smaller REA. Consequently, the typical reduction of the REA in AVH patients could be best explained by stronger interhemispheric gamma-band coupling, given that GBO are known to be critically involved in cortical circuity abnormalities in SCZ^25, 26^. Thus, we expected specifically in AVH patients (1) an altered language lateralization that becomes apparent as reduced REA, and (2) a stronger interhemispheric gamma-band coupling between bilateral auditory cortices.

## Methods

### Participants

This study included 26 patients who met the DSM-IV criteria for SCZ and 26 healthy controls (HC) matched for age, sex, and education. Inclusion criteria were German as a native language, right-handedness, no current presence and/or history of co-morbidities with axis I and II disorders of the DSM, no neurological disorders, no history of alcohol/drug dependence or abuse within three months, an IQ of ≥70, and no hearing damage or tinnitus. Most patients were taking antipsychotic medications, but there was no medication that would have interfered with EEG, neurological or cognitive functions (e.g. Clozapine; Table 1). Psychopathologic symptoms were assessed using the Positive and Negative Syndrome Scale (PANSS)^27^, which was evaluated according to Gaag *et al*.^28^. The individual mental state was examined using the subjective well-being under neuroleptic scale (SWN-K)^29^. The patients were carefully assigned according to the current history of AVH using the positive PANSS item “P3” into two groups (AVH vs. non-AVH patients). The Psychotic Symptom Rating Scales (PSYRATS)^30^ was used to document the phenomenological features of the voices. The group of healthy subjects partly overlapped with the sample of our previous study (4 new subjects were recruited for matching)^15^. This study was approved by the ethical committee of Medical Association Hamburg and was consistent with the principles outlined in an internationally recognized standard for ethical conduct in human research. After participants received a complete description of the experimental procedures, written informed consent was obtained according to the Declaration of Helsinki.

**Table 1:** Demographic and clinical characteristics of subjects: mean and standard deviation (SD) are given for each variable.

Variable	HC (n = 26)	All SCZ (n = 26)	AVH (n = 13)	Non-AVH (n = 13)
	Mean ± SD	Mean ± SD	Mean ± SD	Mean ± SD
Age (years)	32.04 ± 9.28	30.04 ± 9.84	30.85 ± 10.29	29.23 ± 9.71
Gender (male/female)	13/13	14/12	6/7	8/5
Chronic/First episode	n.a.	17/9	8/5	9/4
Smoking status (y/n)*	7/19	17/9	6/7	11/2
Laterality index*	23.74 ± 20.15	9.41 ± 13.56	5.96 ± 8.76	12.86 ± 16.75
Number of errors*	23.69 ± 15.36	40.85 ± 20.06	45.31 ± 21.27	36.38 ± 18.51
Number of LE reports	81.54 ± 19.42	90.69 ± 15.67	92.23 ± 8.98	89.15 ± 20.63
Number of RE reports	135.58 ± 26.97	110 ± 20.08	103.69 ± 17.62	116.31 ± 21.06
Educational level	3.5 ± 0.74	3.35 ± 1.54	3.92 ± 1.7	2.77 ± 1.16
Verbal IQ	110.15 ± 9.16	99.65 ± 15.75	102.62 ± 13.22	96.69 ± 17.97
Response time LE*	3.07 sec ± 0.32	3.50 sec ± 0.8	3.63 sec ± 1.02	3.38 sec ± 0.51
Response time RE*	2.98 sec ± 0.30	3.45 sec ± 0.74	3.57 sec ± 0.95	3.33 sec ± 0.46
Age at onset (years)	n.a.	23.96 ± 8.31	22.30 ± 6.79	24.78 ± 9.72
GAF	n.a.	45.88 ± 16.82	42.08 ± 14.26	49.69 ± 18.84
PANSS scores:	n.a.			
Positive ^a^		18.00 ± 7.20	22.15 ± 1.92	13.84 ± 4.75
Negative		16.69 ± 7.98	18.07 ± 2.97	15.30 ± 6.54
Disorganization		19.73 ± 6.22	19.76 ± 2.97	19.69 ± 8.47
Excitement		14.46 ± 5.27	14.61 ± 5.72	14.30 ± 5.02
Distress		17.80 ± 6.31	18.15 ± 6.81	17.46 ± 6.03
Hallucination (P3) ^a^		2.65 ± 1.76	4.08 ± 1.18	1.00 ± 0.00
Global PSYRATS score	n.a.	n.a.	33.00 ± 8.20	n.a.
Medication dose/CPZ-equivalents	n.a.	339	382	303
Atypical		14	6	8
Both atypical and typical		3	1	2
Atypical + antidepressants		7	4	3
None		2	2	
SWN-K	n.a.	47.91 ± 7.09	50.54 ± 8.65	45.76 ± 4.32

HC, Healthy controls; SCZ, schizophrenia patients; AVH, patients with hallucinations; non-AVH, patients without hallucinations; SD, standard deviation; CPZ, chlorpromazine; n.a., not applicable; RE, right ear; LE, left ear; GAF, Global Assessment of Functioning; PANSS, Positive and negative symptom scale; PSYRATS, Psychotic Symptom Rating Scales; SWN-K, subjective well-being under neuroleptic scale.

*Group ANOVA is significant (*p* < 0.05).

^a^Significant difference between AVH and non-AVH patients(*p* < 0.05).

### Paradigm

The DL task used was the same as in our previous study (for detailed description see ref. 15 and Fig. 1). In brief, six different consonant-vocal syllables (/ba/, /da/, /ka/, /ga/, /pa/, /ta/) were paired and presented simultaneously: one syllable to each ear, resulting in 12 different combinations. Participants were instructed to report after each trial which syllable was perceived best. The response was given via button press with the right (dominant) hand, while a response screen, which appeared right after hearing the syllable pair, showed all six syllables presented in a circular formation. By clicking with the index finger the left mouse button it was possible to navigate through the six answer alternatives and by clicking with the ring finger the right mouse button the selection was confirmed. In order to assess the magnitude of the ear effect, a behavioral laterality index (LI) was calculated for every subject according to the formula: LI = 100 *(RE − LE)/(RE + LE), where RE = number of correct right ear reports and LE = number of correct left ear reports. The scale varies between −100 and +100, with negative values indicating a left-ear advantage (LEA) and positive values indicating a REA.

### EEG recording and functional connectivity analysis

The recording took place in a sound-proof and electrically shielded cabin, while participants listened through closed system headphones (Sennheiser, HAD 200) to the randomly presented 240 syllable pairs at approximately 75 dB. The EEG recordings were conducted with 64 Ag/AgCl electrodes mounted on an elastic cap (ActiCaps, Brain Products, Munich, Germany), including four EOG channels to monitor eye movements, using the Brain Vision Recorder 1.10 (Brain Products, Munich, Germany). Data were recorded with a bandpass (0.1 to 1000 Hz) and digitized at a sampling rate of 1000 Hz. Impedances were kept below 5 KΩ.

Offline processing was carried out using Brain Vision Analyzer 2.0 (Brain Products, Munich, Germany). The data was Butterworth zero phase bandpass filtered from 20 to 120 Hz (IIR, 12 dB/octave) and down-sampled to 500 Hz All channels were re-referenced to common average and FCz was recovered as a regular channel. Epochs with muscle artifacts in any channel were identified by visual inspection and rejected from further analysis. Independent component analysis (ICA) was applied to identify and remove blink, horizontal eye movements, electrocardiographic, muscle and saccadic spike potential (SP) artifacts based on their characteristic topographies, time-courses, and frequency distributions^31^. To control for saccadic SPs in the gamma frequency range^32^ an additional “radial electro-oculogram channel” (REOG) was derived following the procedure described by Keren *et al*.^33^. Subsequently, the artifact-free data was segmented in epochs ranging from −200 to 1848 ms regarding the stimulus onset and sorted as RE or LE reports. Finally, the number of correct-response epochs were exported for further analysis and balanced between LE and RE reports for every subject (matched number of trials for AVH: 68.23 ± 19.43; non-AVH: 68.76 ± 14.79 and HC: 71.19 ± 12.93), with no significant difference in the number of trials between the three groups [*F*_(1,51)_ = 0.208, *p* = 0.81].

Similar to our previous studies^15, 23^, all further analyses were executed with the LORETA KEY software package^34^ investigating functional connectivity computed as “lagged phase synchronization” (LPS)^35^. LPS was assessed within two a priori defined regions-of-interests (ROIs), which have been identified as essential nodes in the AVH network:^36, 37^ right and left primary auditory cortices (PACs/BA41) known to support any type of sound processing, and right and left secondary auditory cortices (SACs/BA42) known to be involved in the processing of complex sounds and speech sounds. The ROIs were defined using the anatomical definitions provided by the eLORETA software based on the Talairach Daemon (see Figure S1). Based on our previous work, where we did not find LPS differences in any of the other frequencies (delta, theta, alpha, beta) as well as an effect in all gamma subbands (30–50 Hz, 50–90 Hz), analysis was focused on the whole gamma-band range (30–100 Hz) and the two conditions: LE and RE report. LPS across all the voxels included in the ROIs was based on the average of all LPS values, which were calculated for the connectivity between every voxel of ROI A and every voxel of ROI B. For all single trials, time-varying frequency analysis was done using a short time Fourier transform (sliding Bartlett-Hann window function) with a window width of 100 ms. LPS was calculated using cross spectra derived from that transformation. In a time frame from 0 to 900 ms after stimulus presentation results were extracted every 100 ms.

Details of EEG electrode placement, the computation methods and its advantages have been provided previously^15, 23^ and can be found also in supplementary material.

### Statistics

First, the patient groups were compared by means of independent t-tests or Mann-Whitney-U tests (depending on normal distribution and corrected for multiple testing) for clinical data using SPSS 21. In order to compare demographic and behavioural data across the three groups (HC, AVH, and non-AVH) one-way ANOVA was used with follow-up Bonferroni post-hoc analysis. Effect sizes were calculated as *r* (for Mann-Whitney-U tests) or partial Eta-squared (*η* ^2^/for one-way ANOVA).

The effects of group, ROIs, condition, and time on gamma synchrony were assessed with a linear mixed-effects model. Linear mixed models have several advantages over traditional repeated-measures designs, as they can accommodate departures from the assumptions of homogeneity of regression slopes and independence, and thus are better suited to model interindividual variability^38, 39^. This model has also been successfully implemented by researchers to analyse EEG data^40–42^. In this study, the model included *Group* (3 levels: HC, AVH, non-AVH), *Time* (10 levels: from 0 to 900 ms in 100 ms steps), *Condition* (2 levels: LE and RE reports), and *ROIs* (2 levels: BA41, BA42) as the fixed-effects factors, Subject as a random-effects factor, and Gamma Synchrony as the dependent variable.

The Spearman’s correlation coefficient (two-tailed, *p* < 0.05) was used to investigate the relation between gamma synchrony (LPS difference between LE and RE reports at 600ms), PANSS hallucination item “P3”, and medication dosage in CPZ equivalents, without applying correction for multiple testing as the analysis was considered exploratory. Bootstrapping was used to calculate 95% confidence intervals (CI) around each significant correlation.

## Results

### Demographic and clinical data

**Figure 2:**
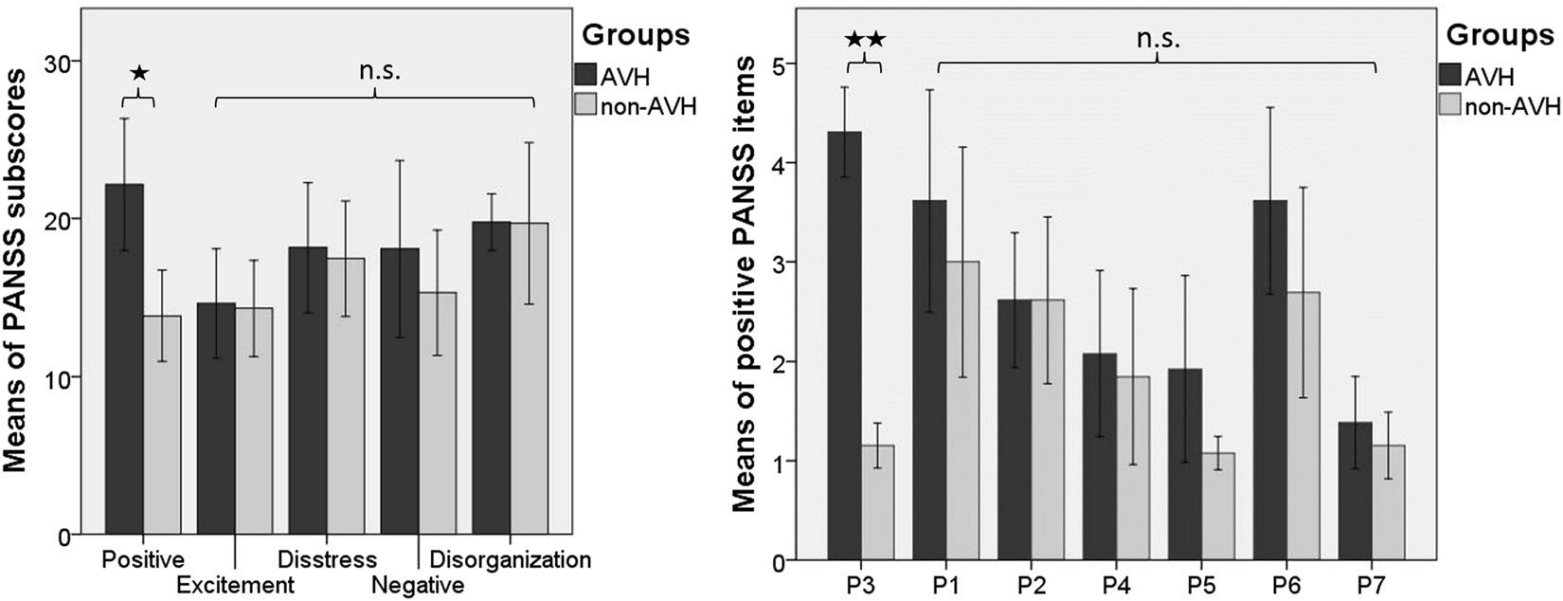
PANSS subscores and positive PANSS items (mean and error bars represent 95% CI). There were significant differences in positive PANSS subscore and the P3 item, which was also used to carefully classify the patients into AVH and non-AVH subgroups (*p < 0.05; **p < 0.01). All other PANSS subcores and positive items were not significantly different. AVH, hallucinating patients; non-AVH, non-hallucinating patients; P1, delusion; P2, conceptual disorganisation; P3, hallucination; P4, excitement; P5, grandiosity; P6, suspiciousness/persecution; P7, hostility.

As shown in Fig. 2, there was a significant difference between AVH and non-AVH patients in positive PANSS scores [z = −2.859, *p* = 0.004, corrected for multiple testing *p* = 0.012, *r* = 0.56], including the item P3 (Hallucinations) [z = −4.683, *p* < 0.001, corrected for multiple testing, *r* = 0.91]. None of the other PANSS subscores (i.e., negative, disorganization, excitement, emotional distress), single positive PANSS items (i.e., P1-P7), or demographic and psychopathology measures, nor chlorpromazine equivalent dose, were significantly different between the two patient groups.

### Dichotic listening task performance

#### Comparison between HC, AVH and non-AVH patients

**Figure 3:**
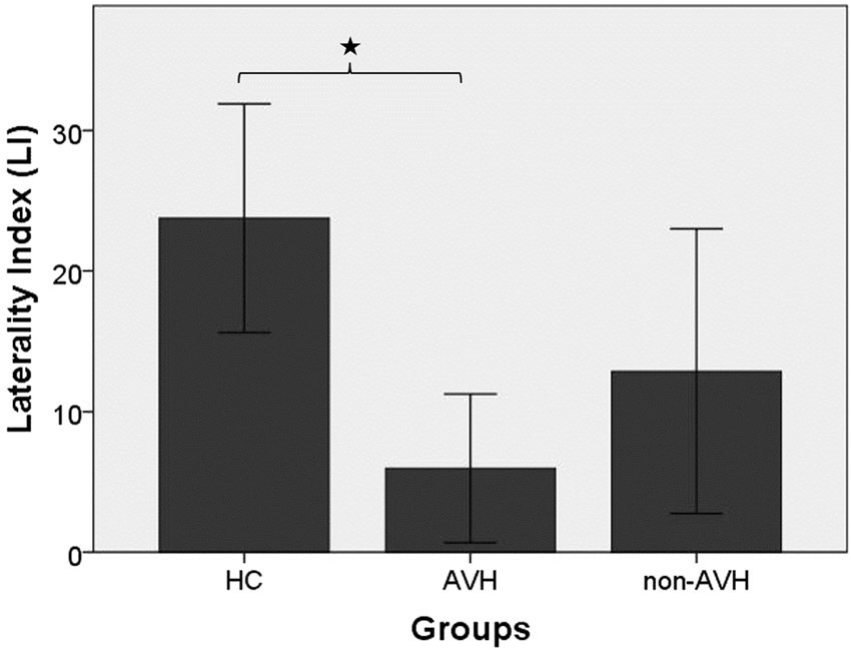
Task performance (mean and error bars represent 95% CI). Significant differences are marked with an asterisk (*p < 0.05). HC, healthy controls; AVH, hallucinating patients; non-AVH, non-hallucinating patients.

There was a significant between-group difference for LI [*F*_(2,51)_ = 5.055, *p* = 0.010, *η* ^2^ = 0.17] with post-hoc analysis indicating a significantly reduced LI in AVH [*p* = 0.011], but not in non-AVH patients [*p* = 0.204; Fig. 3] compared to HC. In addition, there was a significant between-group difference for errors [*F*_(2,51)_ = 6.889, *p* = 0.002, *η* ^2^ = 0.21] and reaction times [RE condition: *F*_(2,51)_ = 4.992, *p* = 0.011, *η* ^2^ = 0.17*;* LE condition: *F*_(2,51)_ = 3.726, *p* = 0.031, *η* ^2^ = 0.13]. Subsequent post-hoc analysis showed a significantly higher number of errors in AVH [HC vs. AVH: *p* = 0.002], but not in non-AVH [*p* = 0.121]. Moreover, AVH patients showed significantly longer reaction times than controls [HC vs. AVH: LE condition: *p* = 0.031; RE condition: *p* = 0.011], while there were no significant differences between HC and non-AVH patients [*p* > 0.2]. Finally, there were no significant differences between AVH and non-AVH patients in LI, reaction times and errors [*p* > 0.59].

### Functional connectivity by means of Lagged Phase Synchronization (LPS)

**Figure 4:**
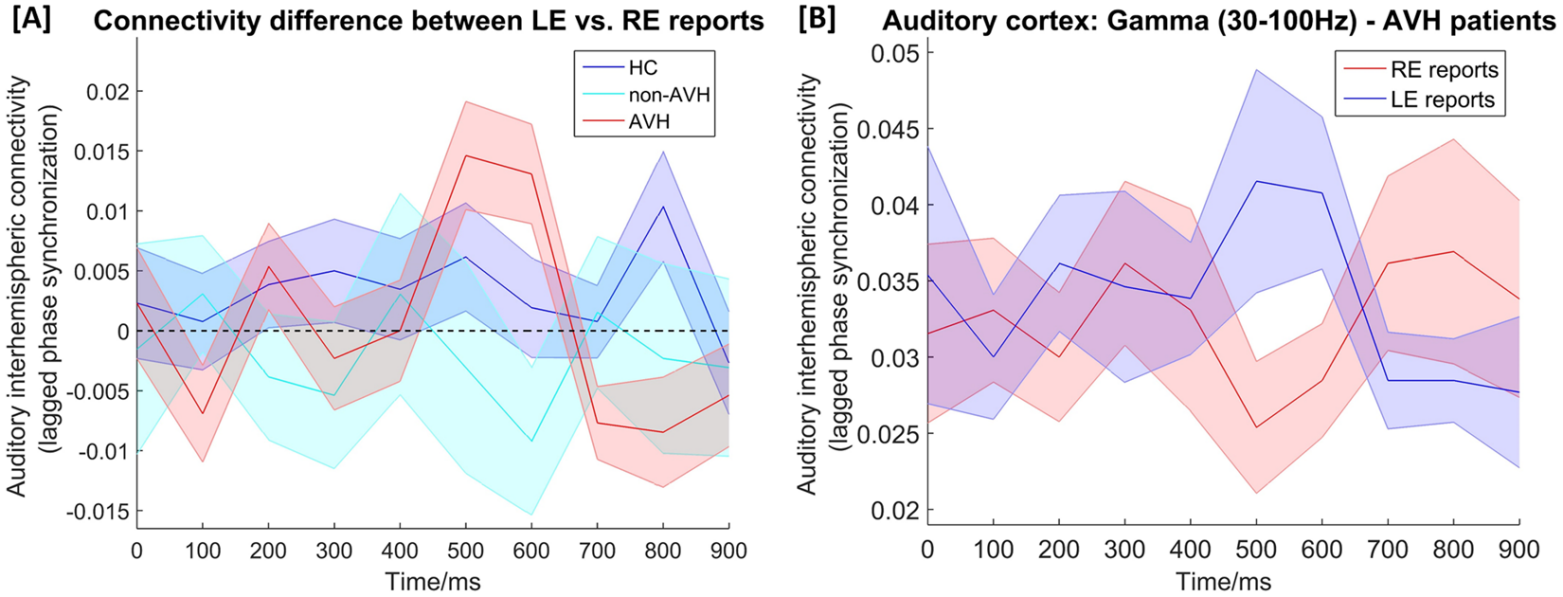
(**A**) Time course of interhemispheric connectivity difference between left ear (LE) and right ear (RE) reports in the gamma-band (30–100 Hz) between bilateral auditory cortices (BA41 and BA42) for AVH, non-AVH and HC participants. Syllable presentation starts at 0 ms and ends around 500 ms. The quality of gamma-band connectivity was found to be significant differently over time across the three groups. (**B**) Time course of interhemispheric gamma-band connectivity during conscious perception of LE or RE syllables between bilateral auditory cortices of hallucinating patients (AVH). Shaded error bars: +/− 1 standard error. HC, healthy controls; AVH, hallucinating patients; non-AVH, non-hallucinating patients; LE, left ear; RE, right ear.

The interaction of *Group x Time x Condition* had a significant effect on *Gamma Synchrony* of both ROIs [*F*_(9,1156.228)_ = 2.250, *p* = 0.002], indicating that there are differences in terms of quality of connectivity over time and conditions across the three groups (Fig. 4A).

In order to further decipher this interaction, each group was examined separately. This analysis revealed in the AVH group that the interaction of *Time x Condition* had a significant effect on *Gamma Synchrony* [*F*_(9,288.215)_ = 2.856, *p* = 0.003], but not in the non-AVH or HC group [p > 0.28]. In the AVH group, subsequent post-hoc pair wise comparison of every time point between *LE and RE reports* revealed greater difference in the gamma synchrony at 600 ms at statistical trend level [*t* = 2.217, *p* = 0.055]. These results indicate that the connectivity difference between LE and RE reports was particularly larger around 600 ms post-stimulus in AVH patients (Fig. 4B).

There was no significant interaction effect of *Time x Condition* in both the non-AVH and HC group, while *Time* had a significant effect on *Gamma Synchrony* in both non-AVH [*F*_(9,289.945)_ = 2.326, *p* = 0.015] and HC [*F*_(9,577.057)_ = 2.202, *p* = 0.021]. Moreover, there was a significant effect of *Condition* on *Gamma Synchrony* in HC [*F*_(1,449.277)_ = 16.323, *p* < 0.0001]. Subsequent post-hoc pair wise comparisons of *LE and RE reports* revealed increased gamma synchrony during conscious perception of LE reports [*t* = 3.389, *p* = 0.002] (see supplementary material), a finding that is in line with our previous results showing that synchronized GBO are crucially involved during transcallosal auditory information transfer in HC. There was no significant effect or interaction effect of ROI in any of the analyses.

### Correlations between gamma-band synchrony and auditory hallucination item

**Figure 5:**
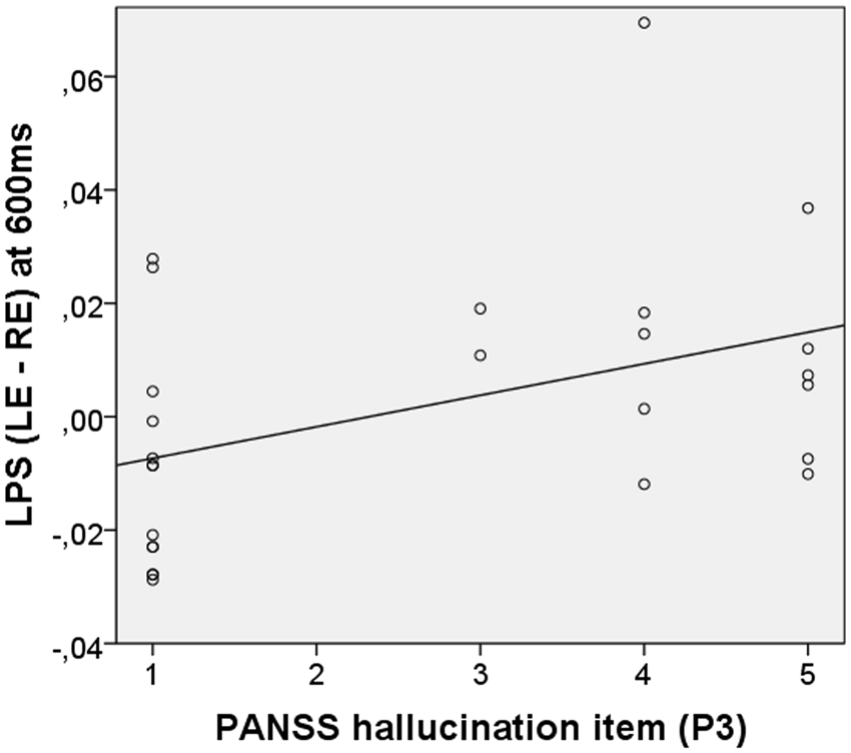
Spearman’s correlations between PANSS hallucination item (P3) and gamma-band synchrony (LPS) between left and right auditory cortices.

A significant positive correlation was observed between gamma-band synchrony and the PANSS hallucination item “P3” [rho = 0.422, *p* = 0.032, bootstrap CI = 0.044–0.699, JZS Bayes factor = 1.48], indicating that a relative stronger gamma-band connectivity between left and right perceived syllables was associated with increased severity of AVH in SCZ patients (Fig. 5). No other PSYRATS items or chlorpromazine equivalent dose revealed significant correlations with behavioral or electrophysiological data.

## Discussion

In the present study we investigated the relationship between functional interhemispheric connectivity in the gamma-band frequency range, conscious auditory perception during DL, and the occurrence of AVH. In accordance to our hypothesis, the major finding was significantly stronger gamma-band connectivity between bilateral auditory cortices during conscious perception of left (versus right) ear syllables in patients with AVH compared to HC and patients without AVH. A significant positive correlation suggested that greater connectivity differences between LE and RE syllables were associated with more severe AVH in SCZ patients. Besides, we found a significantly reduced LateraIity Index (LI) in patients with AVH compared to HC. To the best of our knowledge, this is the first study indicating that the subjective experience of AVH in patients with SCZ is associated with greater gamma-band connectivity alterations during DL and with a reduced REA.

Behaviorally, we found a significantly reduced LI in AVH patients but not in non-AVH patients compared to HC. In previous studies, atypical laterality was observed more often in SCZ than in the general population, which led several authors to infer that there is a genetic link between altered laterality and SCZ^43^. More recent evidence – based on the enormous heterogeneity between patients with SCZ – suggested that the presence of atypical language laterality in SCZ is symptom-dependent, especially with respect to the occurrence of AVH, as both pertain to the same cognitive system (i.e., language)^44^. This is in line with several previous results of DL tasks showing that the current experience of AVH is the determining factor as to whether SCZ patients showed reduced LI or not^3, 4, 45^. In fact, the patient subgroups of this study differed significantly only in terms of PANSS positive subscore including the P3 hallucination item. Accordingly, our and previous findings support the idea that instead of considering SCZ as a homogeneous condition it appears more appropriate to link atypical laterality to specific symptoms, particularly AVH^44^.

Reduced LI in AVH patients is assumed to be indicative of either left auditory cortex dysfunction^2, 45^ and/or interhemispheric miscommunication^3, 4, 11^. The main finding of this study supports the latter by demonstrating that AVH patients show a greater gamma-band phase synchrony difference between LE and RE syllables, thus stronger interhemispheric connectivity around 600 ms post-stimulus between left and right auditory cortices. This finding nicely extends our previous work of HC indicating that synchronized GBO are a key functional mechanism in the transcallosal auditory transfer and play a major role for conscious auditory perception of LE syllables^15^. Neural GBO and their synchronization have been proposed in literature to be a fundamental mechanism that coordinates widely distributed neurons into dynamically formed functional networks, facilitating, for instance, coherent perceptions^16, 46^. If these dynamics are disrupted, it is suggested that pathological states emerge that lead to neuropsychiatric symptoms, such as AVH. In accordance with that, we found a positive correlation between gamma synchrony and the PANSS hallucination item, indicating that a greater connectivity difference between left and right perceived syllables is associated particularly with more severe AVH. Indeed, multiple studies have shown that especially positive symptoms are correlated with increased oscillatory gamma synchrony in circumscribed sensory brain areas in the resting state, and during auditory or visual processing^19, 21, 23, 47, 48^. For instance, it has been shown that phase locking of the 40-Hz auditory steady-state responses – which is generated in the PACs – was correlated positively with total positive symptoms in first-episode patients^49^ as well as with AVH symptoms in a sample of chronic SCZ patients^21^. Also concordant with our finding, presence and subjective intensity of tinnitus – which is similar to AVH an auditory phantom percept – have been shown in MEG studies to be associated with increased GBO^50^ and their synchronization^51^. Thus, former and our own observations suggest that the cortical areas involved in generating auditory phantom percept might be hyperconnected, a hypothesis that is compatible with heightened BOLD responses observed, for instance, during ongoing experience of AVH in the respective PACs^52^. Accordingly, we found enhanced gamma-band phase synchrony between bilateral auditory cortices comprising both PACs.

There are several reasons for altered ability to establish precise synchronization in the gamma-band between bilateral auditory cortices. These involve, for instance, the white matter properties of interhemispheric pathways that subserve phase synchronization^53^. As mentioned above, previous studies using DTI revealed both stronger and decreased interhemispheric auditory pathways in patients with AVH^10, 11^, suggesting a progressive structural decline that might be associated with higher age and longer period of illness. In healthy people with AVH^54^ and patients with tinnitus^55^, stronger interhemispheric connectivity has been observed, similar to early stages of SCZ. Furthermore, emerging evidence proposes a role of excitatory-to-inhibitory balance in maintaining stable perceptual representations, suggesting it may be a plausible model for hallucinations^56^. Thus, the pattern of increased gamma-band coupling in AVH might be considered in terms of the N-methyl-D-aspartate glutamate receptor (NMDAR) hypofunction hypothesis^57^, given that ketamine – a NMDAR antagonist – has been found to elicit in HC both altered GBO^58^ as well as psychotic conditions very similar to the positive symptoms of SCZ, including AVH^59^. Such NMDAR antagonists alter the excitation of parvalbumin-expressing, fast-spiking GABAergic interneurons, which are instrumental in the generation of GBO^60^ and are known to be disturbed in SCZ^61^ as is also the case for NMDAR function^62^. Moreover, a recent MR spectroscopy study revealed significantly reduced glutamate levels in both auditory cortices in SCZ patients compared to HC, while increased levels were found for patients with frequent and severe AVH, relative to patients with less frequent and severe AVH^63^. Thus, one hypothesis might be that AVH arise to some extent from aberrant oscillatory gamma synchrony that is associated with disturbed GABAergic transmission, NMDAR plasticity, glutamate level and hyperactivity in auditory cortices^56^. However, the systematic investigation of relevant synaptic dysfunctions and mechanisms underlying SCZ symptoms and altered GBO has only just begun.

Concerning limitations and strengths of the present study that warrants discussion, the small sample size deserves mention. Further, all but two patient were medicated, but there was no correlation of the neurophysiological parameters with individual chlorpromazine equivalents. Moreover, there was no difference in chlorpromazine equivalents between AVH and non-AVH patients. It is known that altered GBO are also found in untreated patients with SCZ^64^. In addition, EEG-based connectivity analysis has the advantage of measuring direct neuronal activity with an excellent temporal resolution in the millisecond-range (not possible with fMRI), so that the advanced analysis of LPS after source estimation provides an important estimate of functional connectivity between distributed brain regions. The specific strength of this method is that confounding factors such as volume conduction effects are removed and only physiological connectivity information is thought to remain^35, 65^. Of course, one limitation is the relatively low spatial accuracy, although cross validation studies using simultaneous EEG and fMRI have suggested sufficient validity of the LORETA approach in general^66, 67^. It has been shown that the Euclidean distance between EEG- and fMRI-based localizations range between 1 and 2 cm. Thus, our finding of increased gamma-band synchrony between both PAC and SAC (with no difference between ROIs) could be influenced by the low resolution of our source estimate approach. One promising methodological next step providing thus both high spatial and temporal resolution might be the investigation with simultaneously applied EEG-fMRI during DL.

In summary, these results support the hypothesis that AVH are related to interhemispheric miscommunication in the gamma-band range between bilateral auditory cortices, regions critical for auditory perception. Furthermore, our results indicate that altered interhemispheric gamma synchrony is specific to the occurrence of AVH, rather than to SCZ in general. Indeed the observed findings of altered GBO in SCZ patients are complex and suggest that important variables such as the specific symptom loading profile, condition (i.e., resting-state or specific cognitive demands), duration of illness, medication, and clinical status (first-episode, acute exacerbation, chronicity) might affect results and need to be considered in future studies. Moreover, an improved knowledge about GBO-generating mechanisms provides a useful basis for hypothesis-driven analysis of the pathophysiological origins of AVH that may eventually lead to novel insights and treatments. Finally, the implied relation between reduced LI, greater connectivity in favour of LE reports, and severity of AVH, which we observed in our data, suggests altered synchronized GBO as one underlying mechanism of dysfunctional auditory perception in AVH patients.

## Electronic supplementary material

Supplementary Material
