# Supplementary material for: Auditory verbal hallucinations related to altered long-range synchrony of gamma-band oscillations
Source: Sci Rep. 2017 Aug 21;7:8401. doi: 10.1038/s41598-017-09253-7 (PMC5566404; doi:10.1038/s41598-017-09253-7)
Supplement: Supplementary file 1 — Supplementary Material [file 41598_2017_9253_MOESM1_ESM.doc]

1. **Auditory verbal hallucinations related to altered long-range synchrony of gamma-band oscillations.**
2. Saskia Steinmann, Gregor Leicht, Christina Andreou, Nenad Polomac, Christoph Mulert

**SUPPLEMENTARY MATERIAL**

**1. EEG placement**

The recording took place in a sound-proof and electrically shielded cabin. Participants were seated in a slightly reclined chair 1 m apart from a screen while listening through closed system headphones (Sennheiser, HAD 200) to the randomly presented 240 syllable pairs at approximately 75 dB. EEG recordings were conducted at a sampling rate of 1000 Hz with 64 Ag/AgCl electrodes mounted on an elastic cap (ActiCaps, Brain Products, Munich, Germany) using the Brain Vision Recorder 1.10 (Brain Products, Munich, Germany). Electrodes were arranged according to a modified 10/10 system without electrodes at the positions FPz, F9, F10, T9, T10, CP3, CP4, P9, P10, PO7, PO8 and additional electrodes at positions PO9, PO10. Vertical and horizontal eye movement were recorded by two horizontal EOG channels positioned at the outer canthi of the left and right eye and two vertical EOG channels, one below (infraorbital) and one above (supraorbital) the right eye. All electrodes were referenced during recording to ‘FCz’ and ‘AFz’ served as ground. SuperVisc electrode gel (EASYCAP GmbH, Herrsching, Germany) was used to establish contact between the scalp and the electrodes. Impedances were kept below 5 KΩ before starting the recording and checked again at the end.

**2. Lagged phase synchronization (LPS) and eLORETA analysis**

All further analyses were executed with the LORETA KEY software package as provided by Roberto Pascual-Marqui (The KEY Institute for Brain-Mind Research University Hospital Psychiatry, Zurich) at www.uzh.ch/keyinst/LORETA.html. The eLORETA method is a discrete, three-dimensional (3D) distributed, linear, weighted minimum norm inverse solution that has the property of exact localization to test point sources, and no localization bias even in the presence of structured noise.^1^ In the current implementation, computations were made in a realistic head model,^2^ using the MNI152 template^3^ and a 6239-point grid covering cortical areas and the hippocampi at a spatial resolution of 5 mm. As a measure of functional connectivity, we used the non-linear dependence, i.e., lagged phase synchronization (LPS), of intracortical EEG-source estimates. This method measures the similarity of two time series in the frequency domain based on normalized (unit module) Fourier transforms. The LPS measure represents the connectivity between two signals after the instantaneous, zero-lag contribution has been excluded.^4,5^ Such a correction is necessary when using scalp EEG signals or estimated intracranial signals (EEG tomography), because zero-lag connectivity in a given frequency band is often due to non-physiological effects or intrinsic physical artifacts, in particular volume conduction and low spatial resolution.^6,7^ Thus, this measure removes this confounding factor considerably and is thought to contain only physiological connectivity information. The classical phase synchronization, which is highly contaminated by the instantaneous artifactual component, is defined as:

Eq. 1

with:

Eq. 2 $f_{x,y}\left(t,\omega\right)=\frac{1}{N_{R}}\sum_{k=1}^{N_{R}} \left[\frac{x_{k}\left(t,\omega\right)}{\left|x_{k}\left(t,\omega\right)\right|}\right]\left[\frac{y_{k}^{*}\left(t,\omega\right)}{\left|y_{k}\left(t,\omega\right)\right|}\right]$

where *xk* (*t*, ω) and *yk* (*t*, ω) denote the discrete Fourier transforms of the two signals of interest for the *k*-th EEG epoch, *k* =1...*NR*, *NR* being the number of epochs, at time instant *t* and at frequency ω; Re[*c*] and Im[*c*] denote the real and imaginary parts of a complex number *c; c* denotes the modulus; and the superscript “*” denotes complex conjugate. LPS, which statistically partials out the instantaneous component of the total connectivity, is defined as:

Eq. 3

**3. Regions of interest (ROIs)**

**
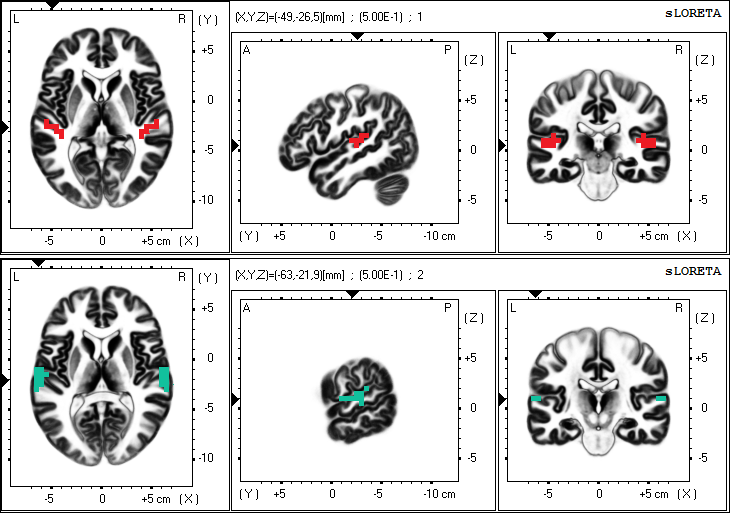
**

*Figure S1.* ROIs within the left and right PACs (red, BA41) and the left and right SACs (green, BA42). BA41-ROI covered a region extended from x: 35 to 55 and -35 to -55, y: -15 to -40, z: 5 to15 and included all voxels of BA41. BA42-ROI covered a region extended from x: 55 to 70 and -55 to -65, y: -10 to -35, z: 5 to 20 and included all voxels of BA42.

**4. Functional connectivity by means of Lagged Phase Synchronization (LPS) between bilateral auditory cortices in 26 healthy controls**

**
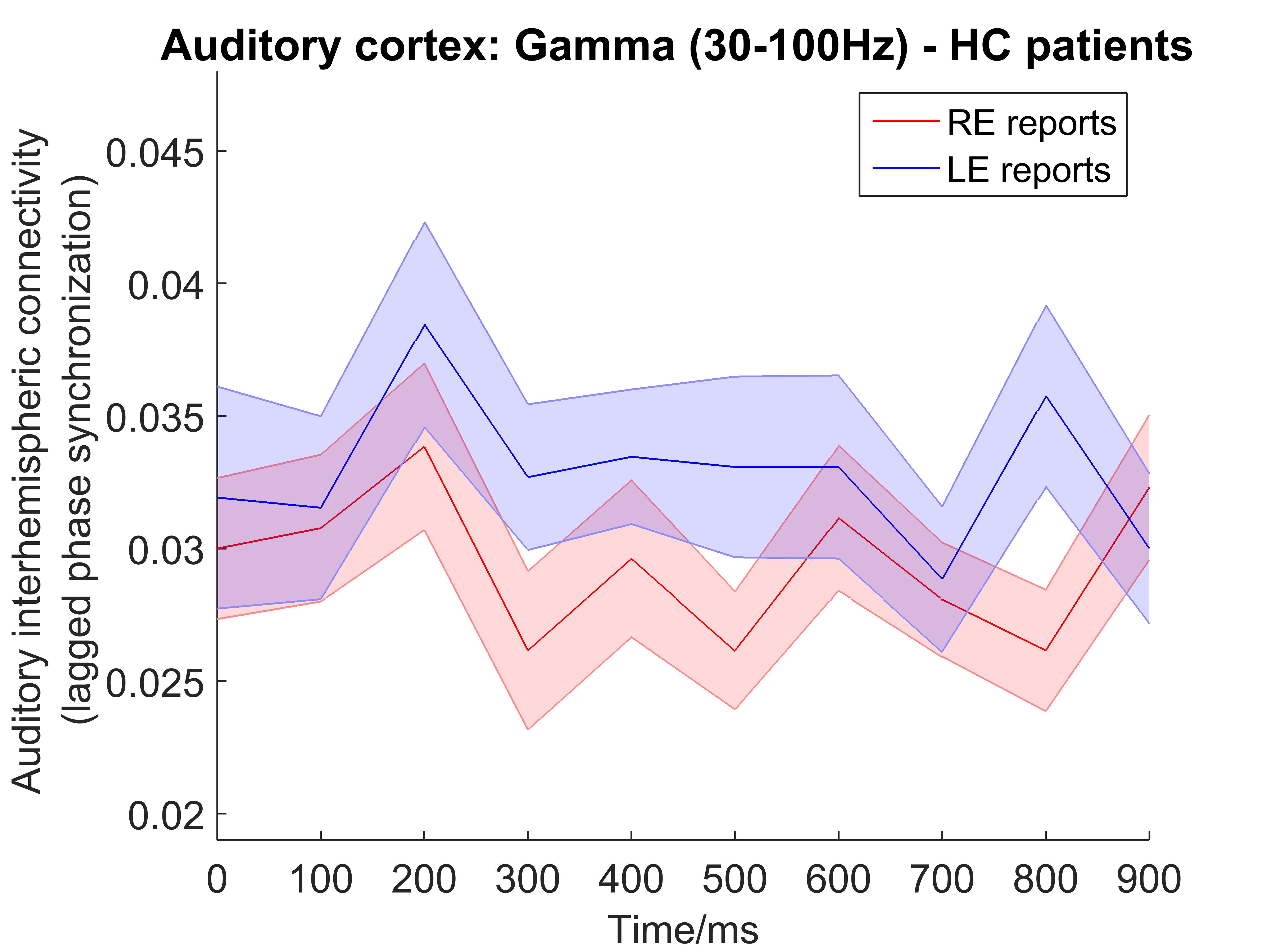
**

*Figure S2.* Time course of interhemispheric gamma-band connectivity during conscious perception of left ear (LE) or right ear (RE) syllables between bilateral auditory cortices (BA41 + BA42) of 26 healthy controls. There was a significant effect of Condition on Gamma Synchrony [*F*(1,449.277) = 16.323, p < .0001]. Subsequent post-hoc pair wise comparisons of LE and RE reports revealed increased gamma synchrony during conscious perception of LE reports [t = 3.389, p = .002], a finding that is in line with our previous results showing that synchronized GBO are crucially involved during transcallosal auditory information transfer. Shaded error bars: +/- 1 standard error

**References**

1 Pascual-Marqui, R. D. Discrete, 3D Distributed, Linear Imaging Methods of Electric Neuronal Activity. Part 1: Exact, Zero Error Localization,. *arXiv:* First published on 17 October 2007, http://arxiv.org/pdf/0710.3341. (2007).

2 Fuchs, M., Kastner, J., Wagner, M., Hawes, S. & Ebersole, J. S. A standardized boundary element method volume conductor model. *Clin Neurophysiol* **113**, 702-712, doi:S1388245702000305 [pii] (2002).

3 Mazziotta, J. *et al.* A probabilistic atlas and reference system for the human brain: International Consortium for Brain Mapping (ICBM). *Philos Trans R Soc Lond B Biol Sci* **356**, 1293-1322, doi:10.1098/rstb.2001.0915 (2001).

4 Pascual-Marqui, R. D. Instantaneous and lagged measurements of linear and nonlinear dependence between groups of multivariate time series: frequency decomposition. *[arXiv: 0711.1455 [stat.ME]* (2007).

5 Pascual-Marqui, R. D. *et al.* Assessing interactions in the brain with exact low-resolution electromagnetic tomography. *Philos Trans A Math Phys Eng Sci* **369**, 3768-3784, (2011).

6 Nolte, G. *et al.* Identifying true brain interaction from EEG data using the imaginary part of coherency. *Clin Neurophysiol* **115**, 2292-2307 (2004).

7 Stam, C. J., Nolte, G. & Daffertshofer, A. Phase lag index: assessment of functional connectivity from multi channel EEG and MEG with diminished bias from common sources. *Hum Brain Mapp* **28**, 1178-1193, doi:10.1002/hbm.20346 (2007).
